# Gathering “tea” – from necessity to connectedness with nature. Local knowledge about wild plant gathering in the Biosphere Reserve Grosses Walsertal (Austria)

**DOI:** 10.1186/1746-4269-8-31

**Published:** 2012-08-13

**Authors:** Susanne Grasser, Christoph Schunko, Christian R Vogl

**Affiliations:** 1 Working Group Knowledge Systems and Innovations, Division of Organic Farming, Department for Sustainable Agricultural Systems, University of Natural Resources and Life Sciences (BOKU), Gregor-Mendel Straße 33, Vienna, 1180, Austria

**Keywords:** Local knowledge, Herbal tea, Wild plant gathering, Regional identity, Revitalisation of tradition

## Abstract

**Background:**

Wild plant gathering is an essential element in livelihood strategies all over the world. However due to changing circumstances in Europe, the reason for gathering has altered from one of necessity in the past to a pleasurable activity today. Wild plant gathering has therefore also received renewed attention as a form of intangible cultural heritage expressing local preferences, habits and man’s relationship with nature.

In the Biosphere Reserve Grosses Walsertal (Austria), local people’s knowledge of the gathering of wild plants and their perception of their own gathering activities are being documented. The focus of this paper is on the uses of herbal teas and the informal guidelines for gathering plants that have been issued by the *Bergtee* (mountain tea) association.

**Methods:**

Thirty-six free-list interviews were conducted with subsequent semi-structured interviews and three focus group meetings held with members of the *Bergtee* association. Participatory observation (gathering and processing plants, mixing and marketing tea) also allowed for greater understanding of what had been reported.

**Results:**

In total, 140 different gathered plant species were listed by respondents. Herbal tea is the most frequently mentioned use. The *Bergtee* association, founded by a young man and two middle-aged women in the valley, is a good example of the link between biological and cultural diversity, with the aim of sharing the biosphere reserve’s natural treasures as well as local plant-related knowledge in the form of herbal tea products. The association’s informal guidelines for gathering reflect people’s attitude to nature: monetary income does not play a major role in gathering plants; instead people’s appreciation of the value of the nature around them is to the fore.

**Conclusions:**

Gathering wild plants can be seen as an expression of people’s regional identity. The conscious appreciation of nature and related local knowledge is crucial for the sustainable conservation and use of the Biosphere Reserve’s resources.

## Background

The gathering of wild plants is an essential element in livelihood strategies all over the world [1,2]. The focus of European ethnobotanical studies on gathering has been on study areas in the Mediterranean region such as Turkey [3], Greece [4], Italy [5,6], Spain and Portugal [7-9], Cyprus [10], Serbia [11], Montenegro [12], Kosovo [13], Bosnia and Herzegovina [14,15]. However there are also a few studies in Central Europe, including Germany [16], Austria [17,18], Switzerland [19] and Poland [20]. These study areas all share a historical shift from agriculture to wage labour which reduced gathering opportunities and activities [3,17,21,22]. Up to the period after the Second World War, people depended on natural resources, and wild plant gathering was an important supplement to daily nutrition in a culture of subsistence [8,18,20] as well as for medicinal needs [17,22]. Nowadays, in many countries the gathering of wild plants is no longer a necessity [22,23].

As local knowledge about wild gathered plant species and their uses is under severe threat as a result of economic globalisation, there is an urgent need to document and safeguard it [24,25], especially in remote areas where this kind of development has not yet led to a complete loss of traditions [26]. It is important to establish a record like this before local knowledge vanishes for good along with the current generation of elderly people [25].

Although local knowledge is vanishing with the elderly, at the same time an interest in this topic can be observed among young and middle-aged people [27]. In a way, plant gathering is becoming fashionable. There are many popular herb books [28-31] focusing on plant species that can be gathered, as well as seminars and courses on plants how to process and use them [17]. In Poland, for example, media publications have led to an increase in the culinary use of some wild plants [20]. In the 1980s, local food producers started to popularise, rediscover and even invent “local products” for selling to tourists [20]. Nowadays, “consumption is determined less by calorie input and more by the pleasure of gathering from the wild, recreating traditional practices and enjoying characteristic flavours” [8].

Wild plant gathering has also received renewed attention as a form of intangible cultural heritage [27,32-35]. This activity is one example of the inextricable link between biodiversity and cultural diversity [36] and reflects symbols of local identities [33]. It is an irreplaceable part of the cultural history of a region [18,37,38] and therefore an expression of people’s local identity and traditions [24,39].

Biosphere reserves are places of high biodiversity and increased awareness of cultural values and identity [40-42]. Their credo is “to use nature without harming it” [43]. In the mission statement of the Biosphere Reserve Grosses Walsertal, the main aim is to raise people’s awareness of cultural values and natural resources as the main basis of livelihood for people in the valley (literally translated from [41]).

It is of interest to see which plant species are gathered and how gathering is perceived by local people who carry out this activity in Grosses Walsertal – the study area being a biosphere reserve and also part of the, in economic terms, highly-developed Austrian province of Vorarlberg. Is gathering vanishing or at risk of being lost? Why do people gather plants today? What knowledge is involved and what is its current image? One association – the *Bergtee* (literally: mountain tea) – dealing with plants and plant knowledge in the Biosphere Reserve Grosses Walsertal has been selected as being representative of local people’s attitudes towards plant gathering. This paper therefore focuses on herbal tea as one example of plant use.

## Methods

### Field site

**Figure 1:**
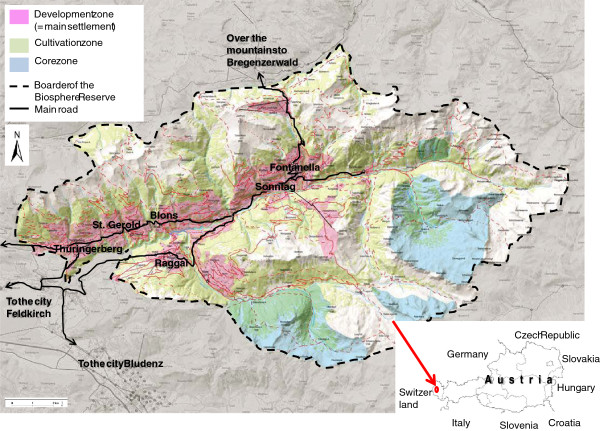
** Map of the Biosphere Reserve Grosses Walsertal in Vorarlberg, Austria (Source:**[28]**, modified).**

Grosses Walsertal is an alpine valley in the centre of Vorarlberg, the westernmost province of Austria (Figure 1). It is a west-southwest, east-northeast-oriented, gorge-like valley without a distinct valley floor, formed by the river Lutz. Covering an area of 192 square kilometers and with a population of 3,400, the valley is very sparsely populated. The dispersed settlements in the valley are mostly situated on the northern (sunny) side. These are the villages of Thüringerberg (880 m a.s.l.), St. Gerold (848 m a.s.l.), Blons (903 m a.s.l.), Sonntag (875 m a.s.l.) and Fontanella (1,145 m a.s.l.). On the southern (shady) side are Raggal (1,015 m a.s.l.) and Marul (976 m a.s.l.) [43]. 

Geologically, Grosses Walsertal is divided into two: the northern part is distinguished by the gentle green mountains of Flysch and the southern part by hills belonging to the Kalkhochalpen (limestone layers). Its altitude ranges from 580 to 2,704 m a.s.l. [43].

The climate in Vorarlberg is typical of central Europe, but relatively cool and with higher precipitation [44]. In Grosses Walsertal, depending on exposure to the sun and altitude, local temperatures and precipitations can vary greatly. The mean annual temperature is 6.7°C (measured at an altitude of 1,140 m a.s.l.). Annual precipitation varies from 1,791 mm per year (at 900 m a.s.l. in Blons) to 1,883 mm per year (at 1,140 m a.s.l. in Fontanella) [45]. The vegetation period across the settlement area of the valley is between 180 and 240 days, again depending on altitude [46]. Due to its geological and morphological variety, the valley is very rich in species [44].

Grosses Walsertal is named after people who emigrated from the Swiss Wallis region in the 13^th^ century. Prior to this, the outer part of the valley had already been inhabited by Rhaeto-Romanic hunters and farmers. The Walser people lived as free peasants and controlled the passes and borders. For a long time, keeping animals for milk and meat formed the sole basis of their existence. Arable farming existed only at self-sufficiency level [44]. One type of agriculture is characteristic of Grosses Walsertal: the *Staffel-*/*Mehrstufenwirtschaft,* a specific kind of transhumance*, i.e.* as the year progresses, farmers move with their livestock (and, especially in the past, with their entire household) to different sites at different elevations: the home farm (winter), the *Maisäß* (elevation 1,300 – 1,700 m a.s.l., spring and autumn) and the *Alp* (elevation > 1,700 m a.s.l., summer) [47].

Due to their remoteness, poverty and subsistence up to the end of the Second World War, *Walser* people used to rely on their local natural resources [48]. For centuries, the people of this remote region were almost cut off entirely. This situation has left its mark: to this day the Walsertal prides itself on a culture and dialect of its own [44].

Gathering plants seems to have a “history” in Grosses Walsertal, even though there is no literature exclusively from this valley to confirm it. Old literature from other nearby regions might also be valid for Grosses Walsertal (*e.g.* from the Montafon [49] or from Switzerland [50]). The botany and local people’s uses of plants in the district of Vorarlberg were documented by Schertler [30], for example, in a popular book full of pictures, stories and recipes.

Local plant knowledge was probably passed on orally or people might have kept handwritten booklets at home. A collection in the Walser dialect was only published in 1996 [51]. This is a collection of statements by local people about plants and other homemade remedies for medicinal purposes. Being part of people’s farming activity, gathering plants was not perceived as something special or extraordinary. However farming and with it the culture of subsistence has changed.

There are now 180 farms in the area, 40% of which are organic [43]. This provides 11.3% of employment, whereas 16.1% of people work in small skilled trade enterprises, 7.7% in tourism and 3.4% in public services. 61.5% have to commute out of the valley for work [52]. Grosses Walsertal became a commuter region from the 1970s onwards [52].

Grosses Walsertal has always been recognised as the poorest part of Vorarlberg [53]. After a varied history, including a huge avalanche disaster in 1954 and the rise of tourism since the 1960s, the valley’s inhabitants were confronted with reorientation and repositioning in the mid-1990s. A strategy was required to give the region a new perspective on economic development [53]. In 2000 Grosses Walsertal was acknowledged as a UNESCO Biosphere Reserve. This led to it being structured into four zones: the core zone (20.9% of the area), the cultivation zone (64.4% of the area), the development zone (13.7% of the area) and the regeneration zone (1.0% of the area) [54].

The Biosphere Reserve’s mission statement declares its aim as being to “raise consciousness of cultural values and natural resources as the main means of livelihood for people in Grosses Walsertal”. One of its main principles is “to be in awe of God and nature and appreciate people’s dignity… A responsible and sparing acquaintance with energy and resources is important to all inhabitants.” (literally translated from [41]). In relation to plant diversity, there are two associations dealing with herbs: the *Bergtee* and *Alchemilla*. The women in these associations use their range of products and services to demonstrate the value of the varied cultural landscape and make an important contribution to adding value in the region [43].

### Free lists, semi-structured interviews, participatory observation

Between July and September 2008, thirty-six interviews were conducted in all six municipalities using free lists and subsequent semi-structured interviews [55,56]. After being given a short introduction to this research project, respondents were asked to list all the plant species gathered in the wild which they know, using the free-listing question: *“Welche Pflanzen fallen dir ein, die hier im Tal wild wachsen und gesammelt werden?”* (literal translation: “What plants can you think of which grow wild in the valley and are gathered?”). In some cases it was necessary to ask further questions (*e.g.* “If you think about the seasons …”), but without ever offering examples of plants so as not to influence the respondents’ answers. When respondents started to run out of ideas, the plants already listed were repeated and respondents asked whether anything else came to mind.

Sampling of respondents was done in the form of snowball sampling [56]. The starting points were members of the *Bergtee* and *Alchemilla* plant associations. In total, 36 respondents, two male and 34 female aged between 27 and 89 years old (average age: 60), were interviewed. Nine of the respondents are involved in the *Bergtee* project and nine in the *Alchemilla* project. Half of the respondents are not involved in any plant-related project, but represent “common” local knowledge about gathering plants in the valley. Twenty-three of the respondents currently run a farm.

A clear expression of consent was requested before each interview. Where applicable, ethical guidelines issued by the International Society of Ethnobiology [57] were followed carefully.

Field notes were taken during the interviews to record the information given and brain protocols completed afterwards. The interviews were also recorded using a digital voice recorder. Photographs of plants and of people gathering and processing plants were taken to provide visual documentation of the information given by local people. Voucher specimens of all plants available during field research and growing in the wild are stored in the herbarium of the University of Natural Resources and Life Sciences in Vienna. Cultivated plants in people’s gardens were not taken as voucher specimens. Plant identification is based on Flora Helvetica [58].

Three meetings were arranged for data collection (focus groups adapted by the authors) with the women involved in the *Bergtee* project: one meeting with women from Raggal, one with women from Thüringerberg, St. Gerold and Blons and one with women from Sonntag and Fontanella, always attended by one or both project leaders. These meetings, usually held by the *Bergtee* association once a year, are used to exchange experiences of plant gathering and use, but also for the leaders to give news of the *Bergtee* project and raise awareness of gathering guidelines. In this case, in 2010, data collection for this project was integrated into the meeting.

Participatory observation was conducted during the whole field research, including plant gathering with local people, helping with agricultural activities (*e.g.* hay making) or processing plants (*e.g.* tea mixing, cooking and ointment or soap making) as well as participating in everyday life and customary festivities (*e.g.* Easter customs, thanksgiving).

An Access database was set up to organise and store the information gathered. Data analysis was carried out in MS Access [59], MS Excel [60] and Anthropac [61]. The interviews were partly transcribed using Express scribe v5.00 [62] and then coded for qualitative analysis.

## Results

First a brief overview is given of the plants reported in the free-list interviews, followed by people’s associations to explain how these listed plants came to be mentioned. As tea is the most frequently mentioned use report, the focus was on the *Bergtee* (mountain tea) association as a very good example of the inextricable link between nature and culture (*biocultural diversity*; [36]). Contact with this association allowed their informal guidelines for plant gathering, which are only transmitted orally, to be documented to show local people’s attitude towards this activity as well as their relationship with nature and natural resource management. This is related to local people’s motivation for plant gathering which has changed over time. The reasons for gathering plants complete the results section with the question “Gathering wild plants – a revitalisation of tradition?” and provide an idea of what prospects might be like in future.

### Gathering wild plants – which plants and why?

**Figure 2:**
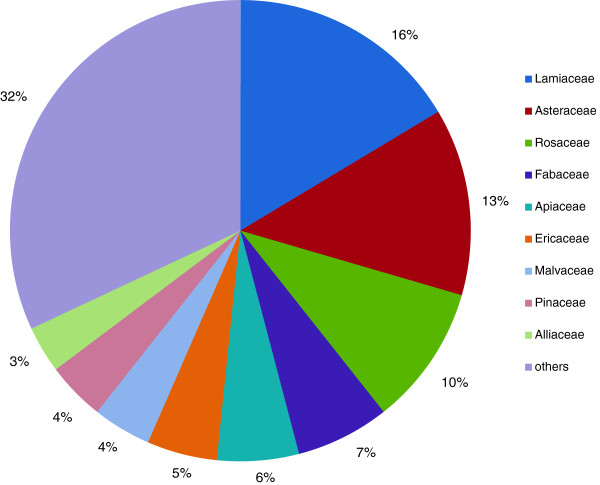
Most quoted plant families in %, frequency of mentions within one plant family>3 (f<3 are summarised as “others”) (n = 36).

The respondents mentioned 892 plant species in all when asked which plants were known to be gathered in the wild in the valley. These reports refer to 140 different plant species belonging to 49 different plant families (families with the most use reports: *Lamiaceae* with 20 plant species followed by *Asteraceae* with 16 and *Rosaceae* with 12). Each respondent listed between 10 and 50 plant species (average: 25 plant species; standard deviation: 8.7) (Figure 2)

The twenty most frequently listed plant species are *Alchemilla alpina*, *Alchemilla vulgaris* L. agg*.*, *Calendula officinalis* L., *Sambucus nigra* L., *Hypericum perforatum* L., *Achillea millefolium* agg., *Rhododendron* sp.*, Urtica dioica* L., *Rubus idaeus* L., *Mentha* sp.*, Primula veris* L./*Primula elatior* (L.)L., *Arnica montana* L., *Plantago lanceolata* L., *Matricaria chamomilla* L., *Picea abies* (L.)H. KARST./*Abies alba* MILL., *Salvia officinalis* L., *Thymus serphyllum* L. agg., *Trifolium pratense* L., *Vaccinium myrtillus* L. and *Taraxacum officinale* agg (Table 1). Each of these twenty plant species was listed by at least 44 percent of respondents.

**Table 1:** The most frequently listed plant species gathered in the wild at the Biosphere Reserve Grosses Walsertal (free lists, n=36, frequency >6). w=growing in the wild, c=cultivated in the garden

**Scientific name**	**German name**	**Family**	**Frequency**	**Mean rank**	**Smith’s S**	Status^*^
*Alchemilla alpina* L. agg.	Silbermantel	Rosaceae	30	8.2	0.58	w
*Alchemilla vulgaris* L. agg.	Frauenmantel	Rosaceae	30	8.8	0.57	w
*Calendula officinalis* L.*	Ringelblume	Asteraceae	29	10.6	0.49	c
*Sambucus nigra* L.	Schwarzer Holunder	Caprifoliaceae	29	13.9	0.38	w, c
*Achillea millefolium* L.	Schafgarbe	Asteraceae	28	10.8	0.47	w
*Hypericum perforatum* L.	Johanniskraut	Hyperiaceae	28	11.8	0.46	w, c
*Rhododendron* sp.**	Alpenrose	Ericaceae	26	11.3	0.41	w
*Urtica dioica* L.	Brennnessel	Urticaceae	23	11.3	0.38	w
*Rubus idaeus* L.*	Himbeere	Rosaceae	22	15.2	0.30	w, c
*Mentha* sp. *	Pfefferminze	Lamiaceae	21	10.7	0.37	c
*Plantago lanceolata* L.	Spitzwegerich	Plantaginaceae	20	10.3	0.35	w
*Arnica montana* L.	Arnika	Asteraceae	20	10.4	0.35	w
*Primula veris* L./*Primula elatior* (L.) L.****	Schlüsselblume	Primulaceae	20	12.0	0.34	w
*Abies alba* MILL./*Picea abies* (L.) H. KARST.*****	Tanne	Pinaceae	19	12.9	0.31	w
*Matricaria chamomilla* L.*	Kamille	Asteraceae	19	15.7	0.23	c
*Thymus serphyllum* L. agg.	Wilder Thymian	Lamiaceae	18	12.7	0.27	w
*Salvia officinalis* L.*	Salbei	Lamiaceae	18	14.6	0.22	c
*Trifolium pratense* L.	Rotklee	Fabaceae	17	12.5	0.26	w
*Taraxacum officinale* WEBER	Löwenzahn	Asteraceae	16	12.4	0.28	w
*Vaccinium myrtillis* L.	Heidelbeere	Ericaceae	16	17.2	0.16	w
*Melissa officinalis* L.*	Zitronen Melisse	Lamiaceae	15	12.5	0.20	c
*Fragaria vesca* L.	Erdbeere	Rosaceae	15	16.2	0.20	w
*Malva neglecta* WALLR.	Käspappel	Malvaceae	15	18.5	0.17	w
*Tussilago farfara* L.	Huflattich	Asteraceae	14	12.9	0.21	w
*Juniperus communis* L.	Wacholder	Cupressaceae	14	13.7	0.19	w
*Equisetum arvense* L.	Acker Schachtelhalm	Equisetaceae	14	15.7	0.20	w
*Tilia* sp.	Linde	Tiliaceae	14	17.4	0.16	w, c
*Bellis perennis* L.	Gänseblümchen	Asteraceae	13	12.1	0.23	w
*Rubus fruticosus* L. agg.	Brombeere	Rosaceae	12	13.8	0.18	w, c
*Ribes nigrum* L.*	Schwarze Johannisbeere	Grossulariaceae	11	13.5	0.15	c
*Peucedanum ostruthium* (L.) W.D.J.KOCH	Meisterwurz	Apiaceae	10	14.0	0.15	w
*Artemisium absinthum* L.	Wermut	Asteraceae	10	15.1	0.13	w, c
*Trifolium repens* L.***	Weissklee	Fabaceae	9	14.4	0.11	w
*Origanum vulgare* L.	Wilder Majoran	Lamiaceae	8	13.1	0.13	w
*Sambucus racemosa* L.	Roter Holunder	Caprifoliaceae	8	18.1	0.09	w
*Pinus mugo* TURRA	Latsche	Pinaceae	8	20.0	0.09	w
*Euphrasia officinalis* L.p.p.	Augentrost	Scrophulariaceae	8	20.0	0.07	w
*Rosmarinus officinalis* L.*	Rosmarin	Lamiaceae	8	20.5	0.08	c
*Rosa* sp.*	Rose	Rosaceae	7	14.8	0.09	c
*Monarda didyma* L.*	Gold Melisse	Lamiaceae	7	16.8	0.06	c
*Symphytum officinale* L.	Beinwell	Boraginaceae	7	18.1	0.09	w
*Lavandula officinalis* Chaix, L. spica L., L. vera*	Lavendel	Lamiaceae	7	20.0	0.06	c

* Respondents were asked for “wild species gathered”. Nevertheless respondents also mention species i) that used to be gathered in the wild and are now grown in gardens and ii) cultivated species.

** Under the term *Rhododendron* sp., *Rh. ferrugineum* and *Rh. hirsutum* are sub-summarised – respondents do not seem to distinguish between these two species in their local name “Alpenrose”. Some respondents use it as the generic term, for some they are just the same plant. Only a few would distinguish between *Rhodedendron ferrugineum* and call it “Alpenrose” and *Rhododendron hirsutum* and call it “Steinrösli”.

*** The local name “Weißklee” is translated with the scientific name *Trifolium repens*. Under this term, *T. montanum*, *T. thalii, T. hybridum* are sub-summarised as respondents do not distinguish between the white flowering clover plants.

**** Only a few respondents distinguished between *Primula elatior* (which they call “Schlüsselblume”) and *Primula veris* (which they call “Himmelschlüssel”). Usually, the local name “Schlüsselblume” and “Himmelschlüssel” were used synonymously.

***** The local name “Tanne” can be either *Abies alba* (“Weißtanne”) or *Picea abies* (“Rottanne”). Some respondents distinguish between these two species; others refer to both plant species using the term “Tanne”.

There is no statistically significant difference in knowledge between the people involved in the *Bergtee* association*,* those involved in *Alchemilla* and “ordinary” people without any involvement in these herb associations.

**Figure 3:**
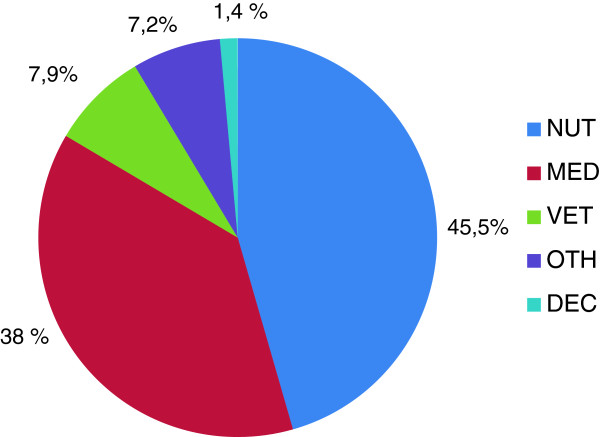
Number of use reports in % sorted by use categories (NUT – nutrition, MED – medicinal, VET – veterinary, DEC – decoration, OTH – others) (n = 36).

The respondents were only asked to list plants, but spontaneously many respondents also mentioned what they use some of these plants for (in total 774 use reports). These use reports were subsequently assigned by the researcher to the following categories: nutrition (NUT), medicinal application (MED), veterinary application (VET), utilisation for decoration (DEC) and other uses (OTH) (Figure 3). The nutrition (NUT) category comprises plants used for eating – *e.g.* eaten raw in salads or added to cakes, biscuits, bread, omelettes, soups, cooked as “spinach” or processed as jam, “honey” (a very thick syrup) or “pesto”. It also includes plants used for drinking, *e.g.* dried for herbal tea, in the form of syrup and juice or added to punch and mixed with alcohol such as liqueur and schnapps or distilled. The medicinal (MED) category includes application forms such as herbal teas for drinking (internal) or bathing (external), oils, ointments for grease and schnapps for rubbing in, but also non-processed raw plants with healing effects (*e.g.* to be eaten or applied fresh on wounds). The veterinary medicine (VET) category includes all medicinal plants also used in veterinary folk medicine as homemade remedies (and most likely also mentioned for humans).

In the decoration (DEC) category the so-called “Alpabtrieb” was mentioned most frequently as a use report. This is a farming tradition and common festivity to celebrate a successful summer on the alpine pasture. Livestock (mainly cattle) and herders are decorated with rosemary (*Rosmarinus officinalis*) and carnations (*Dianthus sp*.), combined with branches of fir (*Abies alba* MILL.*)* or spruce (*Picea abies* (L.)H.KARST.). Juniper (*Juniperus communis* L.) and *Rhododendron* sp. are also used for decoration. During the Alpabtrieb on one specific day in autumn, people and livestock decorated in this way move down from the alpine pastures in the higher regions to their homes.

A few other use reports in this category are the decoration of dishes with nice flowers or leaves. Other uses (OTH) are for fertilizer, insecticides, incense for fumigation or use in herb pillows for example.

A brief description of uses, applications and preparations of all plant species named by more than two respondents shows the diversity of uses practiced by local people in Grosses Walsertal (Table 2). Most of these uses refer to nutritional purposes (NUT category) and plants used for drinking (81 use reports) and eating (60 use reports). This paper contains an overview of these use reports, but the focus is on the use for herbal tea as it is the one most frequently mentioned.

**Table 2:** Plant species gathered in the wild and their uses in the Biosphere Reserve Grosses Walsertal (free lists, n=36, frequency of mentions >2, sorted by frequency f and rank R

**f**	***Scientific name***	**German name (local)**	**Family**	**Organ used**	**Use category**	**Application**	**Preparation**	**MED/VET uses and efficacy (if VET and if specific animals mentioned, animal in brackets)**
30	*Alchemilla alpina*	Silbermäntele	Rosaceae	aerial parts	MED	drink	tea	for women*, incontinence
30	*Alchemilla vulgaris* L. agg.	Frauenmäntele	Rosaceae	aerial parts	MED	drink	tea	for women*, algomenorrhea, regulative, kidney and bladder trouble, incontinence
29	*Calendula officinalis* L.	Ringelblume	Asteraceae	flower	MED/VET	grease	ointment	wounds, varicose veins, afterbirth (cows)
					MED	rubbing in	schnapps	
					MED	bathing	tea	veins
					MED/NUT	drink	tea	blood circulation in feet, stomach and intestine, cancer
					NUT	food	biscuit	
					DEC	decoration	salad	
29	*Sambucus nigra* L.	Holder, Holunder	Caprifoliaceae	flower	MED	application	heated in linen bag over steam	bronchial catarrh, breast inflammation in nursing mothers
					MED/NUT	drink	tea	calmative, diaphoretic, perspiration, cough
					NUT	drink	syrup	
					NUT	food	omelette	
					NUT	food	biscuit	
				leaf	MED/NUT	drink	tea	calmative, diaphoretic, perspiration, cough
				fruit	MED/NUT	drink	juice	strengthening the body’s defences
					MED/NUT	food	jam	cold, cough
					MED/NUT	food	dried	diarrhoea
					NUT	drink	wine	
				bark	MED	grease	ointment	rheumatism
				twig	OTH	instrument	whistle	
				tree	OTH	planting	n.s.	protection against lightning
28	*Achillea millefolium* agg.	Schafgarbe	Asteraceae	aerial parts	MED	bathing	tea	rheumatism
					MED/NUT	drink	tea	anti-inflammatory, for women*, menopause, stomach, circulation, neuritis and pain, cold, blood purification, kidney and bladder trouble
					NUT	drink	liqueur	
					VET	food	tea	anti-inflammatory, antibacterial (bees)
				leaf	NUT	food	“spinach”	(together with *Urtica dioica*)
					NUT	food	salad	
28	*Hypericum perforatum* L.	Johanniskraut	Hyperiaceae	aerial parts	MED	drink	tea	diaphoretic, indigestion, calmative, anti-depressive
					MED/VET	grease	oil	abrasion of vertical column, inflammation, abscess, wounds, erythema, actinodermatitis, earache, wounds (sows); earache (cats)
					MED	grease	ointment	
					OTH	fragrance	herb pillow	calmative
26	*Rhododendron* sp.	Alpenrose	Ericaceae	flower	MED	drink	tea	cough, croakiness, bronchitis, cold, blood purifying
					NUT	food	jam	
23	*Urtica dioica* L.	Brennnessel	Urticaceae	aerial parts	MED/NUT	drink	tea	(blood) purifying
					MED/NUT	food	“spinach”	spring tiredness, iron deficiency
					NUT	food	onion cake	
					NUT	food	herb salt	
					OTH	washing	tea	hair
					OTH	fertiliser	tea	
					OTH	insecticide	tea	
				root	MED	bathing	tea	calmative
					MED	washing	tea	hair
					MED	washing	schnapps	hair
				seed	NUT	food	biscuit	
					NUT	food	bread	
22	*Rubus idaeus* L.	Himbeere	Rosaceae	leaf	MED/NUT	drink	tea	for women*, blood purifying, diuretic
				fruit	MED/NUT	drink	liqueur	circulation, stomach upset
					MED/NUT	drink	schnapps	stomach and intestine
					NUT	food	raw	
					NUT	food	jam	
					NUT	food	cake	
21	*Mentha* sp.	Pfefferminze	Lamiaceae	aerial parts	MED/NUT	drink	tea	stimulating, intensive taste, stomach ache, cooling, cough
20	*Plantago lanceolata* L.	Spitzwegerich	Plantaginaceae	leaf (leaf fibres)	MED	application	raw	earache
					MED	application	raw	wounds, insect bite
					MED/VET	rub in	schnapps	sprain, muscles, rheumatism, venous turgor, joints, back (cows)
					MED/NUT	drink	syrup	cough, lung
					NUT	drink	tea	
20	*Arnica montana* L.	Arnika	Asteraceae	flower	MED	grease	oil	
					MED	grease	ointment	
20	*Primula veris* L.*/Primula elatior* (L.) L.	Schlüsselblume, Himmelschlüssel	Primulaceae	flower	MED	drink	tea	calmative, diuretic, cough
					MED/NUT	food	syrup	cough
19	*Abies alba* MILL.*/Picea abies* (L.) H. KARST.	Tanne	Pinaceae	twig	MED	grease	ointment	blistering ointment
					MED	grease	heated	blistering ointment, drawing salve
					DEC	decoration	decoration of herder’s hat and cattle for “Alpabtrieb”	
				leaf	NUT	drink	tea	
				resin	MED/NUT	drink	schnapps	allergic coryza
				cone	MED	drink	schnapps	allergic coryza
19	*Matricaria chamomilla* L.	Kamille, Opflblümli	Asteraceae	flower	MED/NUT/VET	drink	tea	stomach ache, flatulence, diarrhoea (calves)
					MED	grease	ointment	drawing salve
					OTH	fragrance	herb pillow	calmative
18	*Thymus serpyllum* L. agg.	Thymian, Quendel	Lamiaceae	aerial parts	MED	bathing	tea	cold
					MED/NUT	drink	tea	cold, diabetes, blood purification
					NUT	food	spice	
18	*Salvia officinalis* L.	Salbei	Lamiaceae	leaf	MED	gurgling	tea	sore throat, angina
					MED	food	raw	sore throat
					MED/NUT	drink	tea	stomach, pyelitis
					OTH	fragrance	herb pillow	calmative
					OTH	fumigation	incense	
17	*Trifolium pratense* L.	(Rot)klee	Fabaceae	flower	MED/NUT	drink	tea	cancer
					NUT	food	raw	
16	*Taraxacum officinale* agg.	Schwiblume, Löwenzahn	Asteraceae	leaf	NUT	food	salad	(with potatoes)
					NUT	food	“spinach”	(with *Urtica dioica*)
					NUT	food	onion cake	
				flower	NUT	food	syrup (“honey”)	
					NUT	drink	syrup	
				root	n.s.	n.s.	n.s.	
16	*Vaccinium myrtillus* L.	Heidelbeere	Ericaceae	fruit	MED/NUT/VET	food	raw	costiveness, diarrhoea
					MED/NUT/VET	food	dried	costiveness, diarrhoea
					MED/NUT/VET	food	jam	costiveness, diarrhoea
				leaf	NUT	drink	tea	
15	*Melissa officinalis* L.	Zitronen-Melisse	Lamiaceae	aerial parts	NUT	drink	syrup	
					NUT	drink	tea	
					OTH	fragrance	herb pillow	calmative
				leaf	NUT	food	omelette	
					NUT	drink	punch	
					DEC	decoration	raw	
15	*Fragaria vesca* L.	Erdbeere	Rosaceae	leaf	MED/NUT	drink	tea	blood purifying, diuretic, for women*
				fruit	NUT	food	raw	
15	*Malva neglecta* WALLR.	Käspappel	Malvaceae	leaf	MED	application	raw	suppurative spots
					MED/VET	bathing	tea	suppurative spots, wounds, healing of fractures, supports perfusion, infantile eczema, neurodermatitis, swollen foot (cows)
14	*Tussilago farfara* L.	Huflattich	Asteraceae	leaf	MED	application	raw	wounds
				flowers	MED/NUT	drink	tea	cough
14	*Juniperus communis* L.	Räckholder, Wacholder	Cupressaceae	leaf	MED/NUT	drink	tea	(“Alptee” with *Rhododendron sp.*), diuretic
14	*Equisetum arvense* L.	Acker-Schachtelhalm	Equisetaceae	aerial parts	MED	bathing	tea	back pain, articular gout, kidney and bladder trouble, for women*
					OTH	fertiliser	tea	(with *Urtica dioica)*
14	*Tilia* sp.	Linde	Tiliaceae	flowers	MED/NUT	drink	tea	calmative, diaphoretic, cough, fever
13	*Bellis perennis* L.	Gänseblümchen	Asteraceae	flowers	NUT	decoration	salad	
12	*Rubus fruticosus* agg.	Brombeere	Rosaceae	fruit	NUT	food	raw	
					NUT	food	jam	
11	*Ribes nigrum* L.	Schwarze Johannisbeere	Grossulariaceae	leaf	MED/NUT	drink	schnapps	stomach and intestine
					MED/NUT	drink	juice	vitamins
					NUT	drink	liqueur	
					NUT	drink	tea	
				fruit	NUT	food	jam	
10	*Peucedanum ostruthium* (L.) W.D.J.KOCH	Meisterwurz	Apiaceae	root	MED	grease	ointment	wounds, anti-inflammatory
					MED	chewing	raw or dried	toothache
					MED/NUT	drink	schnapps	circulation
					MED/VET	fumigation	incense	disinfection, navel infection of calves
					VET	grease	ointment	sick foot (cows)
					OTH	carrying in pocket	raw	protection from disease
				root and/or leaf	MED/VET	bath	tea	wounds, claws
					VET	application	raw (crushed)	claw disease (“der Wilder”)
10	*Artemisa absinthium* L.	Wermut	Asteraceae	n.s.	n.s.	n.s.	n.s.	
9	*Trifolium repens* L.	Weißklee	Fabaceae	flower	MED/NUT	drink	tea	for women*
8	*Origanum vulgare* L.	Majoran	Lamiaceae	aerial parts	MED	grease	ointment	cough
					NUT	food	spice	
					NUT	drink	tea	
8	*Sambucus racemosa* L.	Roter Holunder	Caprifoliaceae	fruit	MED/NUT	drink	juice	cold, cough
					MED/NUT	food	jelly	cough
8	*Pinus mugo* TURRA	Latsche	Pinaceae	leaf	MED/NUT	food	syrup (“honey”)	cold, cough
					NUT	drink	liqueur	
					NUT	drink	tea	
					OTH	fumigation	incense	
8	*Euphrasia officinalis* L.p.p.	Augentrost	Scrophulariaceae	flowers	MED	application	raw (with dew)	eyes
				aerial parts	MED	bathing	tea	ophthalmitis
					NUT	drink	tea	
8	*Rosmarinus officinalis*	Rosmarin	Lamiaceae	aerial parts	MED	grease	ointment	(with *Peucedanum osthrutium)*
					MED/NUT	drink	tea	stomach
					NUT	drink	liqueur	
					NUT	food	omelette	
					DEC	decoration	decoration of herder’s hat for “Alpabtrieb”	
7	*Rosa* sp.	Rose	Rosaceae	flower	MED/NUT	drink	tea	for women*
					OTH	grease	ointment	
				fruit	NUT	drink	tea	
7	*Monarda didyma*	Gold-Melisse	Lamiaceae	flower	MED/NUT	drink	tea	heart strengthening
					NUT	drink	syrup	
7	*Symphytum officinale* L.	Beinwell	Boraginaceae	leaf	MED	application	raw (smashed)	bursitis
					NUT	food	salad	
					NUT	food	omelette	
					NUT	drink	tea	
				root	MED/VET	grease	ointment	bursitis, feet, joints, violent pressure, wounds
					MED/VET	bathing	tea	wounds
7	*Lavandula officinalis* Chaix, L. spica L., L. vera	Lavendel	Lamiaceae	flower	MED	bathing	tea	brain strengthening
					OTH	fragrance	herb pillow	calmative
					OTH	hanging up	bunch	moths
					OTH	fumigation	incense	
6	*Betula pendula* ROTH	Birke	Betulaceae	leaf	MED	washing	tea	hair
					MED/NUT	drink	tea	for women*
					NUT	food	salad	
				juice	MED/NUT	drink	raw	spring cure
6	*Verbascum densiflorum* BERTOL.	Königskerze	Scorphulariaceae	flowers	MED	grease	ointment	otitis media, bronchitis, mucous obstruction
					MED	drink	tincture	
					NUT	drink	tea	
6	*Potentilla erecta* (L.) RAEUSCH.	Blutwurz	Rosaceae	root	MED	application	raw	haemostatic
					MED/NUT	drink	schnapps	stomach
					MED/NUT/VET	drink	tea	stomach, diarrhoea
6	*Allium ursinum* L.	Bärlauch	Alliaceae	leaf	NUT	food	soup	
					NUT	food	“spinach”	
					NUT	food	“pesto”	
5	*Lotus corniculatus* L.	Hornklee	Fabaceae	flower	NUT	drink	tea	
5	*Glechoma hederaceae* L.	Gundelrebe	Lamiaceae	aerial parts	NUT	food	soup	
					NUT	food	salad	
					NUT	food	spread	
4	*Anthyllis vulneraria* L.	Wundklee	Fabaceae	flower	NUT	drink	tea	
4	*Chenopodium bonus-henricus* L.	Guter Heinrich	Chenopodiaceae	leaf	NUT	food	“spinach”	
4	*Vaccinium vitis-idaeae* L.	Preiselbeere	Ericaceae	fruit	NUT	food	jam	
4	*Gentiana lutea* L.	Gelber Enzian	Gentianaceae	root	MED/NUT	drink	schnapps	stomach and intestine
4	*Valeriana officinalis* agg.	Baldrian	Valerianaceae	root	MED	drink	tincture	cardiotonic
4	*Sorbus aucuparia* L.	Eberesche	Rosaceae	fruit	NUT	drink	schnapps	
					NUT	food	jam	
4	*Galium odoratum* (L.) SCOP.	Echter Waldmeister	Rubiaceae	aerial parts	MED/NUT	drink	tea	for women*, anti-depressive
					NUT	drink	punch	
4	*Plantago major* L.	Breitwegerich	Plantaginaceae	leaf (leaf fibres)	MED	application	raw	earache
				leaf	MED	application	raw	purulence
4	*Tropaeolum majus*	Kapuzinerkresse	Tropaeolaceae	flower	NUT	food	herb salt	
					NUT	food	salad	
				leaf	NUT	food	salad	
					VET	food	raw	immune system
3	*Carum carvi* L.	Kümmel	Apiaceae	seed	MED/NUT	drink	tea	stomach, flatulence
3	*Verbena officinalis* L.	Eisenkraut	Verbeneceae	aerial parts	MED	drink	tea	wounds
3	*Levisticum officinale* W.D.J.KOCH	Liebstöckl	Apiaceae	aerial parts	NUT	food	spice	
					MED/VET	bathing	tea	open wounds, purulent wounds
					MED/VET	application	raw	open wounds, purulent wounds
3	*Geranium robertianum* L.	Storchschnabel	Geraniaceae	aerial parts	MED	drink	tea	women to become pregnant
					VET	food	raw	animals to become pregnant
				flower	OTH	grease	ointment	
3	*Centaurea cyanus* L.	Kornblume	Asteraceae	flower	NUT	drink	tea	
3	*Primula elatior* (L.) L.	Wald Schlüsselblume	Primulaceae	flower	NUT	drink	tea	
3	*Malus domestica* BORKH.	Apfel	Rosaceae	pod of the fruit	NUT	drink	tea	
3	*Viola tricolor* agg.	Veilchen	Violoceae	flower	NUT	drink	punch	
					OTH	grease	ointment	lips, hands
3	*Allium schoenophrasum subsp. Alpinum* L.	Wilder Schnittlauch	Alliaceae	leaf	NUT	food	raw	
					NUT	food	omelette	
3	*Humulus lupulus* L.	Hopfen	Cannabaceae	fruit	MED/NUT	drink	tea	for women*, cold
					OTH	fragrance	herb pillow	calmative
3	*Satureja hortensis* L.	Bohnenkraut	Lamiaceae	aerial parts	NUT	food	spice	
3	*Petroselinum crispum* (MILL.) FUSS	Petersilie	Apiaceae	aerial parts	MED/NUT	drink	wine	cardiotonic, circulation
3	*Cetraria islandica*	Isländisch Moos		aerial parts	MED	drink	syrup	added to syrup of *Plantago lanceolata*
3	*Pimpinella major* (L.) HUDS.	Bibernell	Apiaceae	root	MED	application	raw (sliced)	between teeth
					MED/VET	bathing	tea	muscles, wounds; claws
				flower	NUT	drink	tea	
3	*Rosa canina* agg.	Hagebutte	Rosaceae	fruit	NUT	drink	tea	
3	*Sedum* sp.	Fette Henne	Crassulaceae	leaf	NUT	food	salad	
					NUT	food	cooked	
3	*Tanacetum vulgare* L.	Rainfarn	Asteraceae	aerial parts	VET	fumigation	incense	disinfection (bee-house)
3	*Capsella bursa-pastoris* (L.) MEDIK.	Hirtentäschel	Brassicaceae	aerial parts	MED	grease	tincture	fracture, inguinal hernia, tissue strengthening
					NUT	drink	tea	
3	*Viscum album* L.	Mistel	Loranthaceae	aerial parts	MED/NUT	drink	tea	regulating blood pressure

In total, the 892 mentions refer to 140 plant species.) *MED* = medicinal, *NUT* = nutritional, *VET* = veterinary, *DEC* = decoration, *OTH* = others. *n.s*. = no statement. Table 2: Plant species gathered in the wild and their uses in the Biosphere Reserve Großes Walsertal (free lists, n = 36, frequency of mentions >2, sorted by frequency f and rank R. In total, the 892 mentions refer to 140 plant species.) *MED* = medicinal, *NUT* = nutritional, *VET* = veterinary, *DEC* = decoration, *OTH* = others. *n.s*. = no statement.

* Respondents often generally said that a plant “is good for women”. This refers to issues such as algomenorrhea, regulation of menstruation, pregnancy and menopause.

### What do people think of when they are asked about plant species gathered in the wild?

#### The link between the wild and gardens

**Figure 4:**
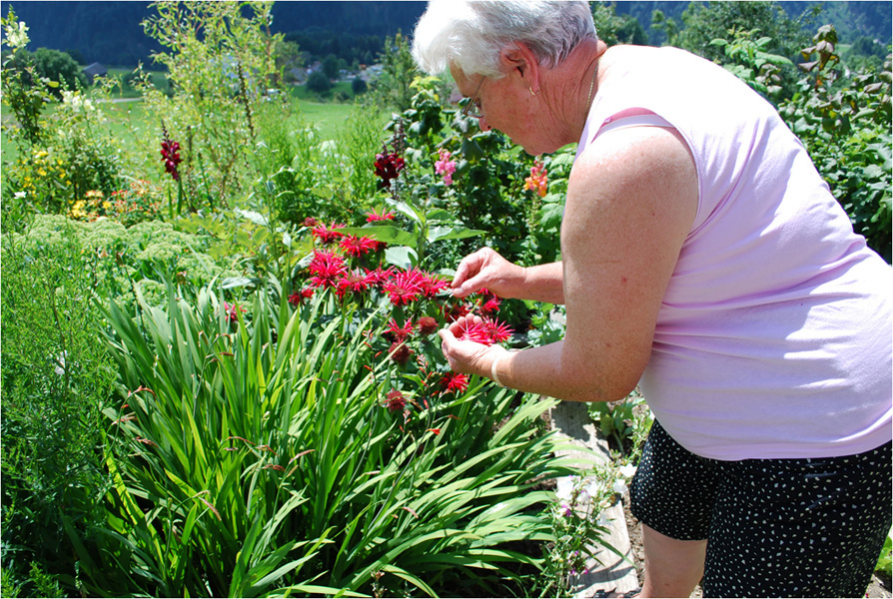
** Woman picking*Monarda dydima*in her garden for tea (Photo: Susanne Grasser).**

Although asked about plant species growing in the wild, almost one third of the plants mentioned grow in people’s gardens (Figure 4). One reason is that some of the plant species mentioned were originally gathered in the wild, but are now cultivated in gardens at home (*e.g. Rubus idaeus*). The respondents think of these species now grown in gardens as wild.

The respondents also seem to link their answers mentally with their uses. By way of example, respondents who mentioned plants collected in the wild for homemade remedies continued to list all the plants they know which are used in homemade remedies, even if they are grown in gardens (*e.g.* ointment from *Hypericum perforatum* L. – growing in the wild – was subsequently linked to *Calendula officinalis* L. which grows in gardens). Other respondents thought of herbal tea plant species (*e.g.* starting with *Alchemilla* sp.*, Thymus serphyllum* L. agg. – which grow in the wild – and then followed by *Melissa officinalis* L. and *Matricaria chamomilla* L.– which grow in gardens).

#### The link between gathering and teas

Plants used for herbal tea were often named first when respondents were asked about plant species gathered in the wild (most use reports in the nutrition category (127 use reports)), within the nutrition category drinking applications (81 use reports), and within this drinking application tea (71 use reports). One reason for this might be local understanding of the term “tea”, expressed in the local turn of phrase in the Walser dialect *“Gö mr gi te sammla”* which is literally translated as “Let’s go and collect tea”). This phrase expresses the idea in the study area that gathering plants means gathering plants for herbal tea.

### *Bergtee* – a community project

Herbal tea has great relevance in the Grosses Walsertal region.

The *Bergtee* (mountain tea) association was founded in 2003 by a young man and two middle-aged women with the idea of bringing together women in the region who are interested in and knowledgeable about gathering plants for herbal tea. The initiative came from the recognition that they have “something valuable” in their valley. They want to share and exchange the “ancient knowledge” held by women in the valley. At the same time, members of this association are expected to be open to “new” ideas: *e.g.* nowadays younger members of this association look up recipes in magazines or on the internet. In addition to knowledge passed on orally, when asked for sources of their knowledge, respondents showed popular herb books and excerpts from newspaper articles (the local newspaper has a “herbal knowledge” column) or talked about courses they had attended. “Everything that works counts”. The members of the association accept knowledge as something that is dynamic.

When the above-mentioned young man in his early twenties went to Vienna to study, he took some tea from the valley with him. Drinking this tea, he remembered his time as a little boy when he spent several summers on an alpine pasture in Grosses Walsertal. The elderly farming women there always sent him to collect “Silbermänetli” (*Alchemilla alpina*) or other herbal plants to take home to his mother at the end of the summer. The women believed that this gathering activity helped against home sickness. This gathering of herbal plants and his childhood memories somehow bound him to his valley. He then shared the herbal mountain tea with his fellow students in the city as an adult and got the idea of marketing it in the city’s tea house where tea from all over the world is sold – but not at that point from Grosses Walsertal. He wanted to share this special and valuable product and so called his mother in the valley to ask if it was possible to send more tea, arrange for women to gather herbal plants and mix blends of *Bergtee*. This finally led to the creation of the *Bergtee* community project.

As well as social events (tea afternoons for exchanging knowledge), the project leaders sell herbal tea in local shops, a few local restaurants and selected teahouses. They guarantee high-quality products made from handpicked herbs from the mountain meadows and alpine pastures which are then air dried and carefully processed. They are not interested in producing large quantities, but rather in understanding and valuing the herbal plants and their effects. They want to pass on this understanding, the way they appreciate and value nature and what it offers their customers (see also: [43]).

The project leaders say their ancestors passed on knowledge about plants from generation to generation and that they knew all about the relevance of the position of the sun, phases of the moon, times for gathering, habitats and air quality. In the *Bergtee* project the women are therefore trying to cultivate this knowledge, pass it on and take it into account when they gather herbal plants for tea.

The project leaders, as well as the women involved who gather plants for the *Bergtee* project, emphasise attentiveness to nature and appreciation of the treasures it provides. Therefore informal guidelines for gathering (and processing) plants have been established and are highlighted every year in an annual meeting at the start of the gathering season. These guidelines are based on experience over the years and are passed on orally. They are like an informal code on good common practice. The project leaders do not control compliance but have trust in the women who gather herbal plants for the *Bergtee* association.

The rules of these informal guidelines start with the beginning of the season. The first flowers that appear (*e.g. Tussilago farfara* L.) are to be left for the bees. When plants are picked, they not all the plants growing at one site should be taken. Where just a few individual plants of a certain species are growing, they should not be picked. Pickers may not walk in the high meadows while gathering (otherwise this will present difficulties for farmers when mowing).

According to their informal guidelines, the best time of day for collecting plants (especially for flowers, *e.g. Calendula officinalis* L.*, Hypericum perforatum* L.*, Trifolium pratense* L.) is around midday when the sun is shining and at its strongest (as the plants themselves are at their peak then as well). Plants should also be allowed two or three days in the sun to “top up” their energy. With both flowers and leaves, it is important only to take dry ones. The morning dew needs to have dried.

Leaves (*e.g. Plantago lanceolata* L.*, Alchemilla vulgaris* L. agg., *Fragaria vesca* L.) have to be inspected carefully. Only healthy ones without holes or any sign of pests or disease may be taken. They have to be gathered while they are still young and fresh, *e.g.* raspberries (*Rubus idaeus* L.) and blackberries (*Rubus fruticosus* L. agg.) are taken before stems become spiky. The leaves of blackcurrants (*Ribes nigrum* L.) should be picked before the fruits ripen.

Roses (*Rosa canina* L.) are taken from gardens and only from plants where pesticides have not been used. Only fresh petals may be picked, not from flowers that have almost withered. Any plant species used for herbal tea has to be gathered solely on unfertilised meadows. A frequent statement – also used for advertising the *Bergtee* – is that “tea herbs should not have heard a car”, meaning that plants are not picked near roads, but higher up in the mountains or on the alpine pastures.

Based on experience, the guidelines state that the constellation of stars plays a role in the quality of the herbal plants and in processing and storing them. Some women have good experiences of gathering under specific zodiac signs, but many women also admit that it is not always possible to bear astrology in mind. These women state that plants have to be picked when the weather is right and they have time. One area of knowledge that has been passed on and is recommended by the project leaders is to pick elder flowers (*Sambucus nigra* L.) at around the full moon. The women find that the flowers retain their yellow colour more, instead of becoming brownish.

They also advise the use of paper or linen bags for gathering. Plants would “sweat” in plastic bags, diminishing their quality. Also for drying purposes, the plants should be well spread out on a white cloth in the dark. No direct sunshine and a bit of a draught allow them to dry evenly. They should be turned every day until they are fully dried which can be recognised by a “crunchy sound” when they are crushed in your hand. The plants are then stored in paper bags which are kept open.

**Figure 5:**
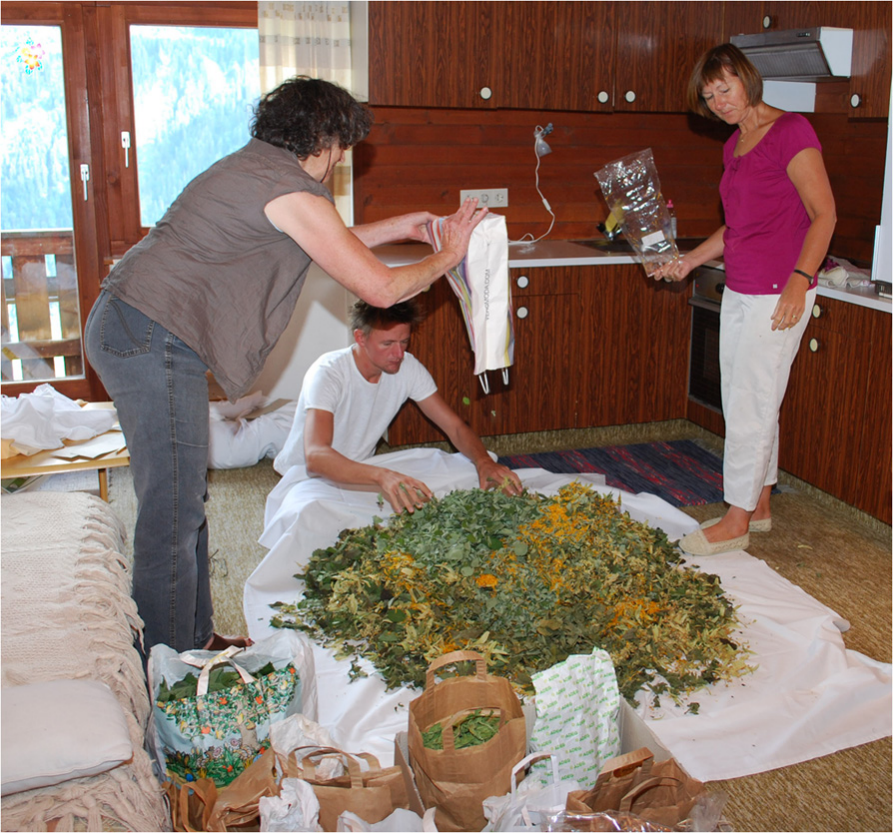
*** Bergtee*project leaders mixing tea herbs (Photo: Susanne Grasser).**

The *Bergtee* project leaders then collect all the gathered plants from several women in the valley to mix different blends for selling (Figure 5). They always combine seven plant species as there is a traditional saying that no illness will come near when you drink a seven-plant blend. However, the project leaders emphasise that under Austrian law they are not allowed to declare their blends to have any medicinal benefits. Sayings are allowed, *e.g.* “Grandmother said ribwort is good for coughs” but nothing more than this. Many women recommend following their own intuition when picking plants: if someone is suffering from an illness, the “right” plant to heal this illness will be growing around them (*e.g.* plenty of flowers of *Tussilago farfara* L. will be found when someone is suffering from a cough). Attention should be paid to instinct: the plant you feel attracted to will also be the one that is right for your needs at the time. In any case, the women mix the herbal plants intuitively to a well-balanced and tasty blend which is never the same twice. Aesthetics are also important, therefore every blend also comprises some colourful flowers (*e.g. Calendulaofficinalis* L., *Monarda didyma*, *Primula veris* L.) to appeal to the eye as well.

**Figure 6:**
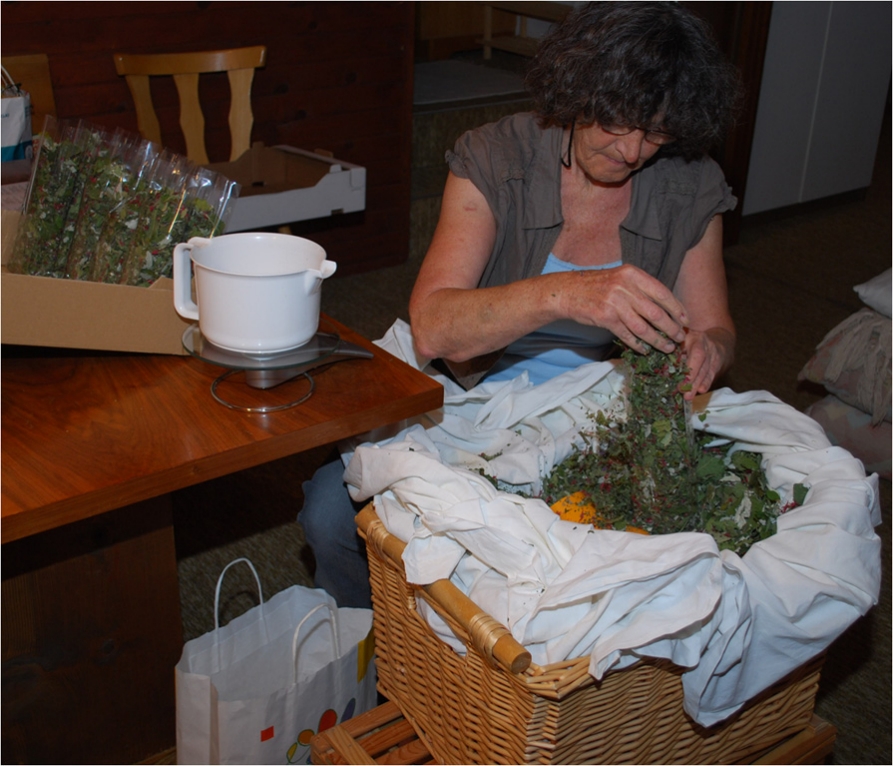
** Packing*Bergtee*tea blends in 30 gram portions (Photo: Susanne Grasser).**

When the plants are packed (Figure 6) – usually in 30 gram portions – attention is again paid “to the current zodiac sign”. Information about which sign is on which day and between which times is looked up in the *“Vorarlberger Schreibkalender”*[63] or any similar “moon calendar”. So called “air signs” (Aquarius, Gemini, Libra) are thought to be best for packing, so that static does not make the dried plants stick to the plastic. For selling purposes, transparent plastic bags are chosen so that people can see what the product looks like. 

Usually, about 40 to 45 kilograms of dried plants per year are gathered for the *Bergtee* association and sold in local shops, a few restaurants and selected tea houses. The women could easily sell more, but say that they just want to gather for as long as they enjoy it. The financial aspect is not a priority. If the packs of tea are sold out, they are sold out and customers have to wait until the harvesting season starts again. Therefore the women of the *Bergtee* project expect that consumers also experience another relationship with and understanding of the cycle of nature. Furthermore, the plant species are all of equal value (financially as well), *i.e.* the project leaders pay their pickers the same price per kilo for *Urtica dioica* L. (growing in large amounts and called a “weed”) as for the tiny petals of *Monarda didyma.*

According to the women, everything in the process – from gathering and drying to packing and storing – has to be done with great diligence. The act of gathering plants always has to be done with pleasure otherwise it is said that the pickers’ own stress and bad energy will flow into the product . Anyone drinking this tea would also drink in the pickers’ stress and anger.

The women emphasise that herbal mountain tea is a product to be enjoyed. When tea is drunk, it provides for a few moments of relaxation. Just a small portion of herbs is enough for a litre of boiled water brewed for ten minutes. Tea should be something sociable – drinking it with other people and using the time for a chat. The *Bergtee* project also brings women together to exchange their experiences as they are always open to learning something old and new. One woman explained that through the *Bergtee* project, she was inspired to start gathering again as she knew about it from her parents but never really practiced it herself until then. Now she greatly enjoys this activity and wants to pass on her knowledge to her own children who like joining her in the alpine pastures. This reflects regional identity and an awareness of the value of surrounding nature. Therefore *Bergtee* is more: *“Es ist kein Produkt – es ist eine Geschichte”* (“It’s not a product - it’s a history”, literally translated [43]).

### Alchemilla – a women’s project

*Alchemilla* is the name of an association of women in the Biosphere Reserve Grosses Walsertal who formed a group in 2006 to deal with the broad issue of herbal plants. Their objective is to show the diversity and value of wild and cultivated plants in the biosphere reserve, pass on their knowledge and traditions and sell products made from the region’s plants. For example, they give courses in balsam or soap making, organise herbal walks and ritual ceremonies and offer a diverse programme. *Alchemilla* is a project by women for women with an economic, ecological and social dimension.

As the focus of this paper is primarily on herbal tea, this project has not been explored in detail. Nevertheless, these *Alchemilla* women are an important catalyst for developing awareness of the value of plant gathering and usage. Their products and approach are rather “new”, introducing various customs and products that are new to the region, while the *Bergtee* association is geared more towards local traditions. As the *Bergtee* was founded first, it might also be seen as steering the development of gathering in the region. In any case, both projects share an appreciation of nature and a respectful exposure to natural resources.

### Gathering wild plants – revitalising tradition?

Respondents explained that in the past gathering plants in the wild was a necessity due to poverty. Nature offered food supplements such as dandelion *(Taraxacum officinalis* agg.*)* added to potato salad or Good King Henry/poor man’s asparagus *(Chenopodium bonus-henricus* L.) and stinging nettle (*Urtica dioica* L.) which were used as “spinach”. Traditional tea blends from the past were also mentioned by respondents. The typical *Alptee* (“alpine tea”), a mixture of lady’s mantle *(Alchemilla alpina*), juniper *(Juniperus communis* L.) and *Alpine rose* (*Rhododendron* sp.), was gathered and drunk on the alpine pastures where many people from the valley spent the summer months with their cattle. These are plants growing anyway which would otherwise not have been affordable. People relied on self-supply for nutrition and medical care. Gathering plants for eating and drinking was as common as making homemade remedies from several plant species (*e.g.* ointments from *Calendula officinalis* L., oil from *Hypericum perforatum* L., syrup from *Abies alba* MILL. and *Picea abies* (L.) H. KARST., bathing with *Malva neglecta* WALLR. and fumigating using *Peucedanum ostruthium* (L.) W.D.J.KOCH).

As people in Grosses Walsertal slowly recovered from the Second World War and products became available in the shops, respondents said they were almost proud then of being able to afford to buy items such as spices like caraway (*Carum carvi* L.) or instant tea bags. Gathering plants was associated with poverty and this was something from which they wanted to be liberated.

It took years – up to the 1970s – before the value of something people gathered and made themselves was appreciated again. The quality of the products themselves became more highly thought of than that of bought products. For one *Bergtee* association member, this perception was enhanced while she was bringing up her children. She could then look back on her own childhood differently. A shift towards connectedness with nature took place.

**Figure 7:**
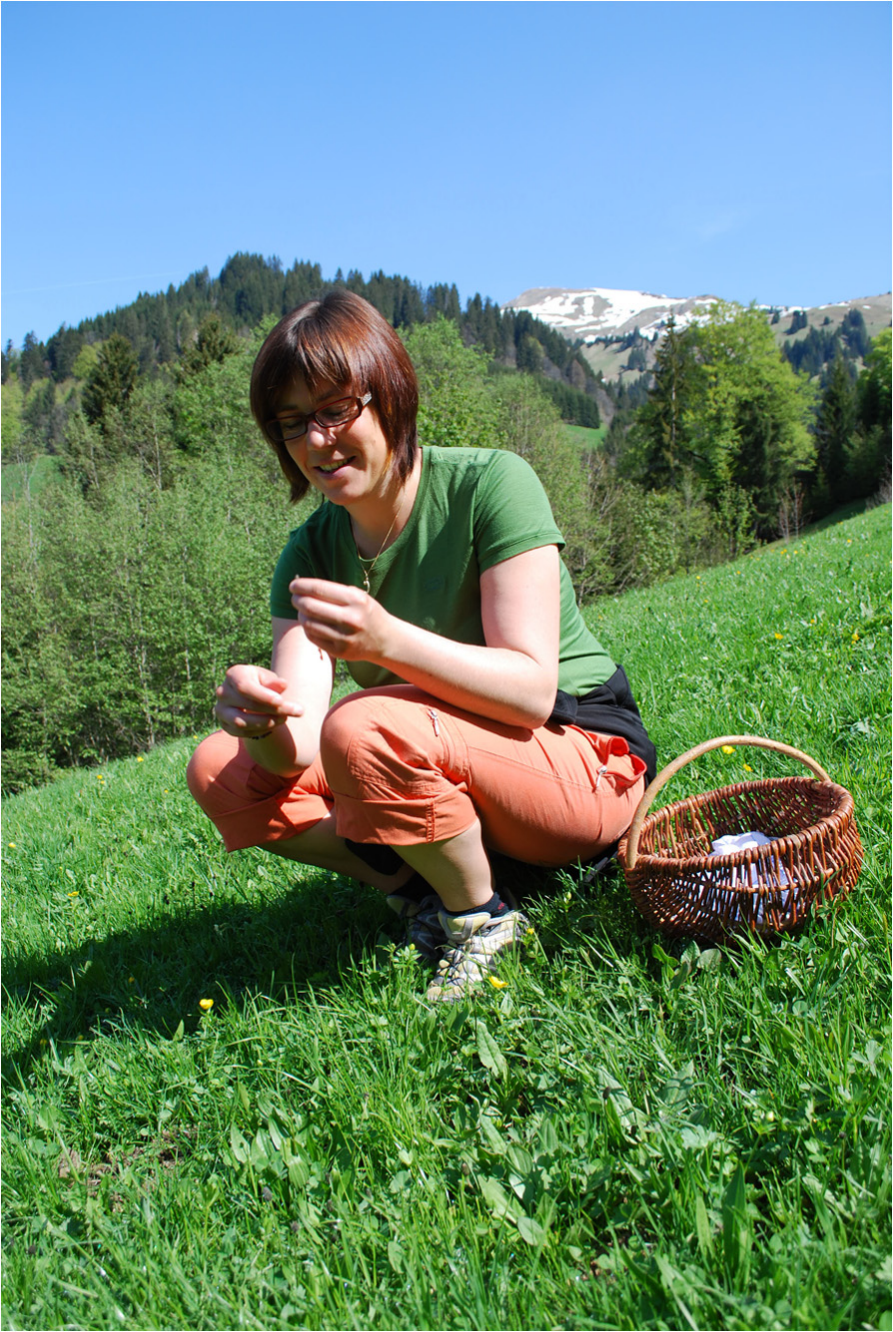
** Woman picking tea herbs (*e.g. Alchemilla vulgaris*,*Taraxacum officinale*,*Plantago lanceolata*) (Photo: Susanne Grasser).**

Now for many of the respondents, the main purpose of collecting plants is simply the pleasure and satisfaction they find in doing it (Figure 7). They enjoy being outdoors, experiencing it as a kind of “time off” in a place where they come to rest and benefit from the calmness of nature while still “doing something valuable”.

Gathering plants also offers an opportunity for a “social event”. Women come together not just to chat, but to “do something useful” (as respondents put it themselves), such as picking berries for example. The respondents would rather meet for a trip to the alpine pastures when it is connected with gathering tea herbs than just go for a walk.

Drinking a cup of tea from the alpine pastures brings back good memories. It allows them to drink in the atmosphere from the past, childhood and beautiful mountains – in this case with good associations rather than still thinking of poverty. There is a certain romance to it. At least for tourists to whom the little tea bags are sold, the product brings with it a sense of holidaying in the beautiful *Grosses Walsertal* mountains.

Reasons for gathering wild plants have therefore changed over time from being something of necessity to allowing a connectedness with nature, enjoyment and pleasure, with socio-economic factors influencing this activity considerably. Therefore, gathering herbal plants for tea – such as by the two women and young man who founded the *Bergtee* project – can be perceived as revitalising tradition.

## Discussion

Plants gathered in Grosses Walsertal are comparable to those that appear in other European studies. Those most similar are the free-listed plant species from Eastern Tyrol, Austria [17], where both vegetation and culture are comparable. Nevertheless, the order (frequency, rank and Smith’s salience) differs. Some plant species are mentioned noticeably less frequently in Grosses Walsertal than in Eastern Tyrol, *e.g. Vaccinium vitis-idaea* L. (mentioned by 81% of respondents in Eastern Tyrol) occurs in lists supplied by Walser people just four times (11%), *Cetraria islandica* (mentioned by 44% of respondents in Eastern Tyrol) just three times (8%). These plants are less widespread in the alpine regions of Grosses Walsertal. This result supports the proposition that the more common a plant species is in an area, the greater the probability of its popular use [64].

Results from Italy [65] regarding the plant varieties gathered are also comparable to those gathered in Grosses Walsertal, but the importance of each single plant (*e.g.* expressed through frequency of mentions) differs between the two regions. While *Alchemilla millefolium* agg.*, Arnica montana* L., *Malva neglecta* WALLR*., Rubus idaeus* L., *Taraxacum officinalis* agg.*, Urtica dioica* L. and *Vaccinium myrtillus* L. are among the most frequently mentioned plant species in Italy and in the Austrian Grosses Walsertal, *Alchemilla vulgaris* L. agg., *Hypericum perforatum* L.*, Sambucus nigra* L.*, Tussilago farfara* L. – included in the most frequently mentioned plant species in the Austrian Grosses Walsertal – are taxa quoted in the Italian study by fewer than 10% of respondents [65]. On the other hand, *Tanacetum vulgare* L.– among the taxa quoted by at least 40% of respondents in Italy – is mentioned by just three of the Austrian respondents (8%). *Peucedanum ostruthium* (L.) W.D.J.KOCH for veterinary folk medicine – among the taxa quoted by more than 40% of respondents in Italy – has a lower value in the Austrian Grosses Walsertal, quoted by just 26% of respondents. *Fragaria vesca* L., *Juniperus communis* L., *Plantago lanceolata* L. and *Thymus* sp. are quoted more often in Grosses Walsertal; *Vaccinium vitis-idaea* L. and *Viola odorata* L. are quoted more often in Italy. Indeed, there are other plants that do not occur in both lists.

Plant species also used in Bulgaria [66] include *Urtica dioica* L.*, Rosa* sp. *Rubus idaeus* L., *Matricaria chamomilla* L. and *Thymus* sp., with many other plants similar to the presented results. In the Mediterranean region, plants such as *Thymus* sp., *Sambucus nigra* L., *Mentha* sp.*, Melissa officinalis* L., *Taraxacum officinalis* agg., *Urtica dioica* L. [26,27,67] are similar to those mentioned in Grosses Walsertal.

It is striking that people in Grosses Walsertal do not mention mushrooms which are however mentioned as a source of wild edible plants in other studies on gathering [7,18]. They do occur in the region and in participatory observation were also detected as being gathered, but seem to have no “history of gathering” in the valley. The mention of *Alchemilla alpina* and *Alchemilla vulgaris* L. agg. at the top of the list in Grosses Walsertal is noteworthy as they are found in just a few studies elsewhere [12,17,65,68-70] (Poland until the 19^th^ century [20]). Respondents refer to the “traditional alpine tea” when they mention *Alchemilla* sp. Alpine pasturing was a traditional method of livestock management and, to a certain extent, is still practiced in the valley. Gathering plants therefore also reflects people’s ways of life: they gather near to where they live [7].

In Grosses Walsertal, the families with the most plant species mentioned are *Lamiaceae, Asteraceae* and *Rosaceae*. This is almost similar to studies conducted for example in Spain (*Asteraceae, Lamiaceae, Fabaceae, Rosaceae*[7,35,71] and Palestine [72]. *“*Té” in Spain refers to 70 different plant species, the most frequent families being *Asteracea* and *Lamiaceae*[67].

As in Grosses Walsertal, the most common use reports elsewhere are as medicine and in food preparation (*e.g.* Eastern Tyrol, Austria: [17], Bulgaria: [66], Spain: [7]). Plants falling along the food-medicine continuum [73] take up a large part of the species list [3,4,19]. Most of the non-crop edible plants and those consumed in drinks have medicinal usage (Spain: 77.3% in [7]). Most frequently applied remedies are teas made from leaves or flowers [17]. In Spain tea (*té*) is also not primarily associated with *Camellia sinensis*, but rather with herbal tea plants [67] – the same as in Grosses Walsertal, where people even use the term “collecting tea” instead of “collecting herbal plants”.

Even just by looking at the titles of papers by other authors, it is obvious that the focus on ethnobotanical knowledge in Europe is on the alimentary and medicinal use of plants. Other use categories are far less represented, *e.g.* use reports for dyeing [74] or in the “domestic, ludic, agropastoral, magic/medicinal, religious, handicraft or magic/ritual/propitiatory” categories [25]. Similarly, fewer use reports in these categories were given in Grosses Walsertal, summarised here as “others” – *e.g.* the “Pfannenfrusi”, a little bunch of dried twigs of *Calluna vulgaris*, which was used in the past to clean pans. Plants gathered for firewood and materials for tools and shelters did not appear in the free lists in Grosses Walsertal. As clearly stated, in this region the idea of gathering is related more to herbal teas than to the scientific concept of wild versus cultivated. It seems that this is the reason why plants used for firewood *etc.* were not mentioned, since these technological aspects are far removed from local people’s associations with the term “plant gathering”.

Compared with “earlier literature” [30,49-51] the interviews did not reveal any totally unknown or surprising results. There seems to be no special “Walser knowledge” that is known and practiced only by people in Grosses Walsertal, but rather common plant knowledge spread over the region.

The fact that respondents also listed plants they cultivated in their gardens has also occurred in other studies, *e.g.* Spain: [7,9,75], Bulgaria [66] and Austria [17]. Christanell [17] argues that this can be explained by a reflection of the history of plant management in the area. Plants which are no longer easily available have been moved slowly into nearby habitats and become cultivated in people’s gardens [17]. This may also be true for Grosses Walsertal. One respondent explicitly stated that she dug up a root of *Peucedanum ostruthium* (L.) W.D.J.KOCH from the Alpine pasture and planted it in her own garden because she was “too old to climb up the mountain anymore”. Nevertheless, we believe that the mention of many plant species (almost one third) grown in people’s gardens is more a psychological matter in terms of associations made by respondents when they were asked to name plant species gathered in the wild. When people thought of herbal plant species or plants they use for tea blends, they simply ended up listing all the plants they use, no matter where these plants are growing. For their work on plants traditionally gathered in the Basque Country, Mendez-Baceta *et al.*[9] also decided to include reports of all species that were referred to by informants as “wild” in their concept of the term, independently of considerations concerning their potential management. The local terms mostly include native species growing in their natural habitat, but sometimes also managed or even promoted by planting their seeds. There are also domesticated species that grew in the area, both cultivated and in the wild. So it was impossible to distinguish between spontaneous or sown species. It is not the botanical or scientific concept of “wild” that counts, but rather local people’s own perception and what they associate with this term.

In many of the sites investigated, the activity of plant gathering, use and management seems to be predominantly done by women [17,76].

Guidelines for gathering plants are not particularly well documented – at least not in ethnobotanical scientific literature. However it is still important to present these informal guidelines as they also reflect people’s attitude towards nature and the utilisation of nature’s products. While in other countries (*e.g.* Bulgaria: [66]) sustainable collecting methods are not practiced, because people (usually working in companies) do not feel connected to the area and so just pick as much as possible, people in Grosses Walsertal use their environment with great respect and gratitude. It is noteworthy just how much respondents emphasised the importance of the sustainable use of natural resources. It is assumed that this is less due to the fact that Grosses Walsertal is a biosphere reserve than to people’s regional identity and appreciation of nature (see also: [47]).

In Bulgaria, for instance, the financial aspects of gathering plants are to the fore [66]. In contrast, making an income from gathered and processed plants is not the main motivation of people in Grosses Walsertal. They primarily gather plants for joy. It is an expression of being connected to nature (see also: [17]). This motivation has developed from a negative perception of wild plant consumption in the past – as a symbol of poverty (see also: [20,75]) – to a very positive connotation nowadays. In some countries, it is also linked to tourist activities (*e.g.* Poland: [20] and Spain: [75]) which has allowed for a revitalisation of wild plant gathering and their use in traditional cuisine as an expression of cultural heritage. It can also be used as marketing for the Biosphere Reserve Grosses Walsertal [43]. However, the strengthening of regional identity outwards (*e.g.* through marketing the Biosphere Reserve) also consolidates the inward identity and awareness of the value of the nature around people. Therefore, wild plant gathering in Grosses Walsertal is not a vanishing knowledge at risk of being lost forever. Instead it is undergoing a process in which traditions are being revitalised.

Gathering plants is something many people seem to do in Grosses Walsertal. Nevertheless, the response of many people in the interviews was: “I don’t know anything special. It’s not worth your while asking me about it”. This expresses a lack of awareness of the value of their knowledge and practice. The belief however is that the foundation of the *Bergtee* association has raised awareness and furthered the exchange of knowledge. It is said that “Walser people do not talk very much” (as one respondent explained) – they are not used to talking about what they do. The *Bergtee* association provides an opportunity for coming together to chat and discuss people’s experiences so that they can all learn from one other. The *Bergtee* association provides an incentive and motivation for gathering plants. However, perhaps it is not so much the gathering activity that has changed through the *Bergtee* association, but rather the exchange of knowledge and awareness of its value.

## Conclusions

**Figure 8:**
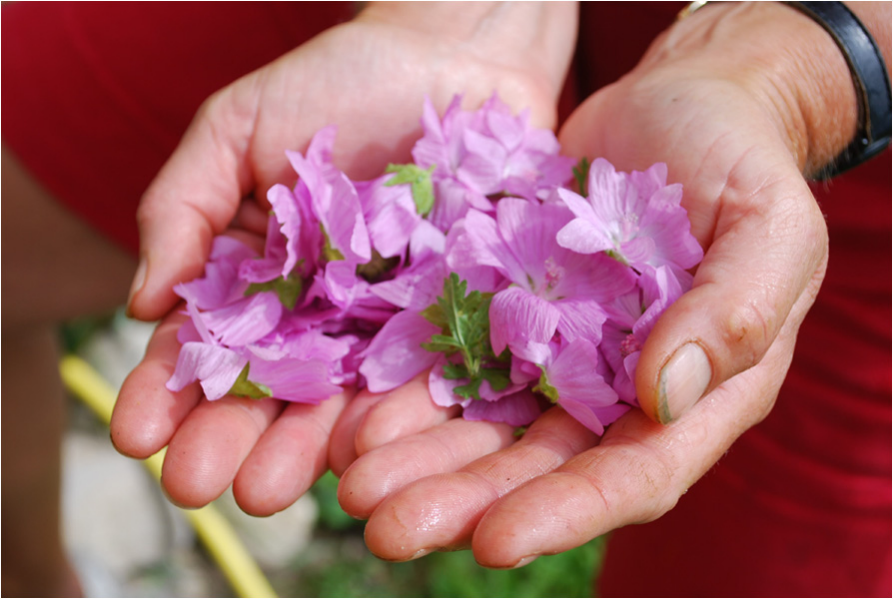
*** Malva sylvestris*for herbal tea (Photo: Susanne Grasser).**

Gathering of wild plant species is a common practice in Grosses Walsertal. While in the past it was seen as a necessity due to poverty, it is now becoming fashionable again as a pleasurable activity. The shift in reasons for gathering, from necessity to connectivity with nature and the appreciation of homemade products, revitalises the gathering activity and use of plant species gathered in the wild. The *Bergtee* (mountain tea) association is one example in the Biosphere Reserve Grosses Walsertal that shows the inextricable link between nature and culture. The marketing of plants gathered in the wild for herbal tea projects people’s regional identity outwards. However, even more important is local people’s own connectedness with the nature around them for a sustainable use of natural resources (Figure 8). The exchange of local knowledge and experiences among the women who gather plants for the *Bergtee* association sustains their customs and passes on this intangible cultural heritage. The request is that the Biosphere Reserve’s management keeps supporting initiatives like this to reinforce people’s awareness of the value of their own local knowledge, their experience and practice and the appreciation of their own habits as an expression of their regional identity.

## Competing interests

The authors declare that they have no competing interests.

## Authors’ contributions

SG designed the methods approach, carried out field work, composed the literature review and drafted the manuscript. CS conducted quantitative data analysis and supplemented the draft. CRV assisted greatly in all stages of this study. All authors read and approved the final manuscript.
